# Prevalence of Alpha Thalassemia in Microcytic Anemia: a Tertiary Care Experience from North India

**DOI:** 10.4084/MJHID.2015.004

**Published:** 2015-01-01

**Authors:** Monica Sharma, Sanjay Pandey, Ravi Ranjan, Tulika Seth, Renu Saxena

**Affiliations:** Department of Hematology, AIIMS, New Delhi.

## Abstract

**Introduction:**

Cases with microcytosis not responding adequately to iron supplementation are diagnostic dilemma and have been reported to harbor alpha (α) thalassemia mutations. The aim of this study was to determine the common α globin gene deletions in cases with microcytic anemia.

**Methods:**

Fifty four patients selected (22 females and 32 males) had microcytic anemia (MCV < 80 fl, Hb <12gm/dl) with raised TRBC (> 5M/mm3) but normal Hb HPLC. They had either low or normal Transferrin Saturation (TS). Gap-PCR for four common α-gene deletions (-α^3.7^, -α^4.2^, - -α^SA^ and --α^SEA^) was done.

**Results:**

Out of the total fifty-four cases nineteen (35.2%) cases were found to have α gene mutations; Three homozygous and sixteen heterozygous cases including -α^3.7^ deletions and a single case of -- α ^SA^ ; but no -α^4.2^ and –^SEA^ mutations were found.

**Conclusion:**

α gene mutations can confound iron deficiency anemia, but no RBC indices, or a discriminant function can identify it is presence Molecular studies have to be resorted to. Gap PCR for common α thalassemia mutation including –α ^SA^ should be done even in the face of low iron stores in subjects who respond incompletely to iron supplementation.

## Introduction

Iron deficiency anemia (IDA), β thalassemia trait (βTT) and anemia of chronic disease (ACD) are common causes of microcytosis that can be diagnosed accurately by Iron studies and Hb HPLC respectively. However, when HbA2 level is normal or low along with normal or low serum iron studies, microcytosis can be a diagnostic dilemma. Walford et al. and Pearson et al.^1^,^2^ had long back suggested that such cases could be harboring α thalassemia mutation.

The α-thalassemia syndromes are among the most common single-gene disorders with more than 20% of the world population to be a carrier of some form of α–thalassemia, as estimated by The World Health Organization (WHO).^3^–^5^ In India also it is common, and the gene frequency of occurrence is appreciably higher than that of βTT.^6^ Deletion - α ^3.7^ is the commonest reported genotype which exists in α + (-α/αα) milder form.^7^,^8^ Most alpha thalassemia cases in India have been reported among tribal populations^8^,^9^ with βTT and other hemoglobinopathies but its co-existence with IDA has never been studied.^7^,^10^–^13^ Iron deficiency is highly prevalent in India and with the reportedly high frequency of α thalassemia the likelihood of the two conditions coexisting can be expected to be high. The Discrimination between microcytosis due to the two conditions is not only clinically significant but is often difficult even more so when they coexist. No Discriminant function or RBC Indices can indicate the presence of α thalassemia in the subjects with or without IDA.^14^–^16^ Molecular studies most commonly deletion-specific gap-PCR have to be resorted to for detection of the common α^0^- and α-thalassemia deletions in such cases when the Hemoglobin or microcytosis does not improve appropriately after iron supplementation.

The mutation spectrum for each population group is distinct characterized by a small number of founder mutations that reflect the predominant mutant alleles in carriers and affected individuals.^17^,^18^ This helps in designing the gap PCR assay individually or in multiplex panels. One of the most popular multiplex assays covers seven deletions, specifically the SEA, FIL, MED-I, THAI, 20.5, 3.7, and 4.2 deletions.^19^–^22^ Other molecular studies though not readily available are multiplex ligation-dependent probe amplification (MLPA)/microarray analysis, allele-specific assays and α-globin gene resequencing for detection of common, rare, and private point mutations.

Based on previous studies in India in our laboratory, gap PCR for - α ^3.7^, -α ^4.2^,- - α ^SA^ and - - α ^SEA^deletions are done.

Microcytic hypochromic anemia is the hallmark feature of α thalassemia, and the degree of microcytosis is directly proportional to the number of alpha genes deleted, thus, reflecting the rate of imbalance between α- and β-chain expression.^23^,^24^ Apart from few studies,^15^, ^25^ there is a paucity of population-based studies on MCV in α thalassemia observing the effect of its variation in alpha genes deletions & also it is an alteration with the coexistent IDA.

This study was undertaken to identify and highlight the presence of common α thalassemia deletions in cases of microcytic anemia and its interaction with IDA.

## Material and Methods

### Patients

Fifty-four Patients, attending Hematology outpatient department, All India Institute of Medical Sciences, New Delhi were included in the study that was approved by institutional ethical committee. Duration of the study was 1.5 years between 2010 and 2011. The cases included had Hemoglobin <12 gm/dl, MCV<80fl and TRBC > 4.5 M/mm^3^. Only cases with standard Hb HPLC showing low or normal HbA2 (< 3.5 %) were taken to exclude βTT/Hemoglobinopathy.

### Sampling

3 ml blood in EDTA for CBC and Hb HPLC and 1 ml blood in 3.2% sodium citrate for DNA studies was obtained after taking signed consent from the patients.

### Hematological work up

Hematological parameters were analyzed by automated cell counter (XT1800i, Sysmex Transasia). Quantification of HbA2 was performed by High Performance Liquid Chromatography (Hb-HPLC, Bio-Rad variant- β thalassemia short program). Serum Iron studies were performed by standard laboratory methods and transferrin saturation (TS) <16% was taken to be iron-deficient.

### Detection of the α thalassemia mutations

Genomic DNA was prepared from peripheral blood by the standard phenol-chloroform extraction method. Deletion mutations were characterized by Gap-PCR. Detection for single deletion - α ^3.7^ and -α ^4.2^ Baysal et al.^26^ **- - α** **^SA^** deletion Shahji et al.,^5^ and **- - α** **^SEA^** Chang et.al. was done.^27^

### Follow-up

Though it was intended to be a cross-sectional study, the Follow-up data on Iron therapy available were evaluated in 27 (54%) cases.

### Statistical analysis

The following parameters were assessed: Hemoglobin, MCV, TRBC, RDW-CV, TS and HbA2. Mutation positive and negative groups were compared. Statistical analysis was carried out using the statistical package SPSS version,^15^ and an independent sample *t-*test was used for comparison of hematological parameters. Statistical significance was assessed as a test with p ≤ 0.05. The hematological parameters of three alpha thalassemia mutations, the four groups based on mutation status and TS </> 16 % were also compared, but the size in each group was too small for obtaining a valid statistical comparison.

## Results

### Patient characteristics

Of the 54 cases included in the study, 32 (57.4%) were males and 22 (42.6%) females and the median age was 19.5 years (13 months – 62 years). There were four pediatric cases (<18 years) in alpha positive genotype group and seven in wild alpha genotype group. The subjects were, for the most, anemic (median Hemoglobin was 9.6 g/dl (5.1–12g/dl), with RBC showing microcytosis (median MCV63.4fl (48–79 fl) and iron deficiency (median TS 10.2% (7.6–19.2%). They had TRBC median 5.2M/mm3 (4.5–6.2M/mm3), median RDWCV 19.3 %( 14–29%) and median HbA2 2.3 %( 1.6–2.8%).

### Characteristics of alpha thalassemia subjects

**Figure 1:**
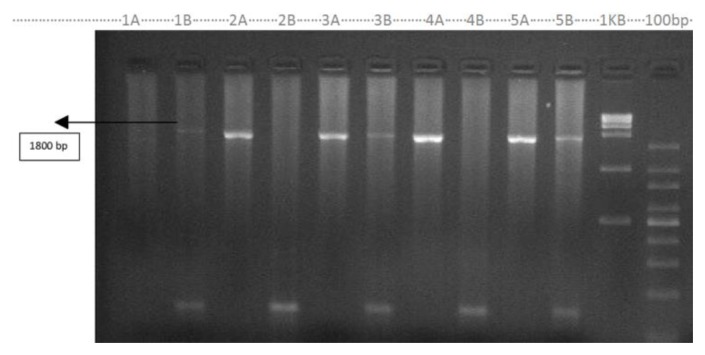
α-3.7 kb mutations. (Lane 1A, 2A,3A, 4A, and 5A are normal set and lane 1B, 2B, 3B, 4B and 5B are mutant set). Lane 1AB patient homozygous, 2AB normal, 3AB heterozygous, 4AB normal and 5AB Heterozygous. Primer A+C (A) normal and A+ B(B) mutant are amplified in separate tubes because their product have same size of1.8 kb - Baysal et al.^26^

**Figure 2:**
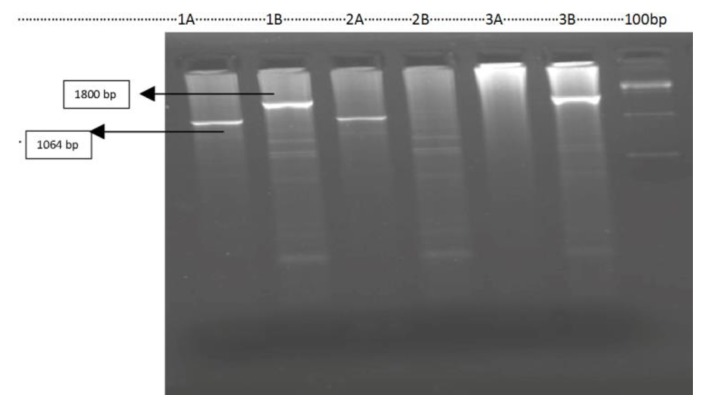
α—SA Mutations. (Lane 1A, 2A,3A are mutant set and lane 1B, 2B, 3B are normal set). Lane 1AB heterozygous, 2AB homozygous and 3AB is normal. Amplification done according to Shahji et al.^5^

Nineteen out of 54 (35.2 %) cases showed α thalassemia deletions and three genotypes were seen on gel electrophoresis. Fifteen were –α^3.7^ heterozygous, three were α ^3.7^ homozygous and one was - - α ^SA^ double deletion (Figure 1 and 2). Comparative hematological parameters of mutation-positive and mutation-negative cases are given in table 1. The subjects had presented chiefly for work up for long standing anemia or detected incidentally and few for nonimmune hydrops. The median hemoglobin was 10.5 g/dl(5.9–11.9g/dl) across the α thalassemia genotypes. The median MCV was significantly lower in the three homozygous with α ^3.7^ deletions, median 56.2fl (56–72.4fl), compared to the median, 68.9fl (47.6–80fl) (29.6%), of the subjects -α^3.7^deletion heterozygous and to the value, 71.6fl, of the one subject (1.9%) –with α ^SA^ thalassemia.

There were high RDW-CV and low %TS indicating coexisting IDA in 10 of the 19 mutation-positive cases. On comparing the hematological parameters of α thalassemia mutation-positive and negative cases (Table 1). The mean Hemoglobin and MCV were better in the mutation-positive cases while the mean RDW-CV was higher in the mutation-negative group (Hemoglobin, MCV p=0.161, p=0.151, RDW-CV and TS p=0.040, 0.001 respectively). Interestingly 28/35(80%) mutation negative cases showed raised TRBC (Table 2) suggesting that a high >5M/mm3 TRBC may not always be thalassemia.

### Effect of coexisting iron deficiency on α thalassemia RBC indices (Table 3)

The median Hemoglobin, MCV, and HbA2 were lower in both the iron deficient groups 1and three, but the values were relatively much lower in the mutation-negative group 1which was predominantly IDA. TRBC was high in both the groups. Between groups 2 & 4 which were both iron sufficient (i.e. TS > 16%) the median Hemoglobin, MCV, TRBC, and HbA2 were lower in group 4 (mutation positive) compared to group2. The RDW-CV was comparable indicating the coexisting IDA in both the groups. This shows that MCV is lower in α thalassemia compared to Normal. Follow up on Iron was available in 27(54.00%) cases. Response was seen predominantly (63.1%) in group 1 as expected but 36.8% in this group also failed to respond no details about the compliance or cause of refractoriness was available. Subjects of Group 2 did not require iron, but they needed to be investigated for other underlying Hemoglobinopathy, which was beyond the scope of this study. In mutation-positive group 3(50%) patients showed no or inadequate response to iron therapy while individuals in group 4 had % TS >16%and did not need iron supplementation. The family history too was significant only in individuals of the group 4.

## Discussion

Fifty-four subjects with microcytosis but with normal Hb HPLC and normal or low Iron studies were evaluated for the presence of common α-thalassemia deletion mutations. Microcytosis was the defining criteria in the study, but it is also important to remember that normal RBC indices do not rule out α thalassemia carrier.^28^ In the current study with gap, PCR α thalassemia could be detected in nearly 1/3^rd^ (35.2%-19/54cases) of the microcytic cases. These results agree with 50% α-thalassemia cases reported in non-anemic microcytosis cases by Borges et al.^29^ in southeastern Brazilian population and the other studies in European or European-derived populations who also have reported α-thalassemia trait in 25%–80% of non-anemic subjects with microcytosis without iron deficiency.^30^,^31^ As reported in various Indian studies -α^3.7^ deletion was the commonest determinant in the study seen predominantly in the heterozygous state (31.7% of microcytic patients)..^7^,^11^ Interestingly a single case of-- α ^SA^ was detected incidentally.^5^ Though 5% and 3.33% cases of -α ^4.2^ , -- α ^SEA^ deletion respectively have been reported in Indian subjects by Sarkar et al.^32^ but none was seen in the study which could have been due to the difference in the ethnic group which in this study was largely North Indian. This also could explain the high frequency of α-thalassemia cases observed even when only microcytic subjects were investigated, and uncommon α thalassemia deletions and point mutations could not be done. This is concurrent with the observations of K Ghosh et al who have reported highest prevalence in the Punjabis.^7^

Phenotypically all the 19 α thalassemia cases were very similar presenting with only mild anemia. None of the cases had jaundice or gall stone disease. Family history was positive in four individuals. Microcytosis was the defining criteria and was most pronounced in the three (5.6%) -α^3.7^ deletion homozygous individuals compared to (29.6%)-α^3.7^ deletion heterozygous and one (1.9%) -- α ^SA^ thalassemia subjects. Though insufficient in number, the findings are largely in concordance with previous reports, where microcytosis has been explained on the basis of α-gene number.^29^,^32^–^36^ IDA causes the Indices to be much lower than in α thalassemia and may have high TRBC in pediatric group and after recent iron supplementation. 1,2,37,38 A report on alpha thalassemia with anemia in children’s revealed it should be considered differential diagnosis.^39^ On comparing the hematological parameters within the four groups, group 1( IDA cases) had the most pronounced microcytosis even more than the cases with IDA coexistent with α thalassemia mutation (group 3) which had more than that of α thalassemia cases (group 4).^1^ (MCV group1 <group3< group4 < group2). The subjects of Group 3 also had much lower RBC indices than that of group 4 which was akin to normal individuals. In conclusion, Using low Hemoglobin & MCV, raised TRBC, normal Hb A2 and deletion specific gap- PCR for common alpha thalassemia mutations (including - -α ^SA^), nearly 1/3 (35.2%) of the 54 cases of microcytic anemia with or without IDA could be typed in the study. Finding of IDA should not be a deterrent for the screening of a coexisting α thalassemia mutation, which is particularly relevant in a country like India where both α thalassemia (in some geographical areas) and IDA occur with high frequency. Larger population studies, including other less common α thalassemia mutations, are required not only to identify the population group specific mutation spectrum but also to observe the interplaying of the two conditions on the RBC indices. In fact red cell indices could differentiate or indicate the coexistence of the two conditions. Until then molecular studies (gap-PCR) should be done for the detection of common α thalassemia mutations especially when the Hemoglobin or microcytosis does not improve appropriately on iron supplementation this can be useful in the countries where α thalassemia is not prevalent because as a consequence of population migrations alpha thalassemia has acquired a truly global distribution.

## Figures and Tables

**Table 1:** The baseline hematological profile & TS% of mutation positive & negative cases (n=54)

Hematological variables	Mutation positive cases n =19 (Mean ±SD)	Mutation negative cases n =35 (Mean ±SD)	p value
Hemoglobin(g/dl)	9.90 **±** 2.15	9.10 **±** 1.85	0.161
MCV (fl)	66.70 **±** 9.66	63.20 **±**7.66	0.151
TRBC (M/mm^3^)	5.20 **±** 0.51	5.40 **±** 0.42	0.129
RDW CV (%)	18.50 **±** 3.99	20.60 **±** 3.11	0.040
HbA 2(%)	2.40 **±** 0.34	2.30 **±** 0.29	0.072
TS (%)	13.50 **±** 3.92	10.40 **±** 2.47	0.001

**Table 2:** TS% and TRBC between mutation positive and negative case

	Mutation	positive	Mutation	negative
	n= 19		n= 35	
	TRBC< 5M	TRBC>5M	TRBC< 5M	TRBC>5M
	**06 (31.57%)**	**13 (68.42%)**	**07 (20%)**	**28 (80.0%)**
**TS <16 %**	04 (66.66%)	06 (46.15%)	07 (100%)	25(89.28%)
**TS >16 %**	02 (33.33%)	07 (53.84%)	00	03 (8.57%)

**Table 3:** Effect of iron deficiency on Hematological parameters with and without α thalassemia mutation

Groups		Hb	MCV	TRBC	RDW CV	HbA2
**1.Mutation neg+TS<16% n =32**	**Median-range**	8.9 (05.1–12)	62.0 (48.1–78.8)	5.20 (4.92–6.21)	20.4 (15.5–28.8)	2.2 (1.7–2.7)
**2.Mutation neg+TS>16% n =3**	**Median-range**	11.9 (10.50–12)	70.1 (68.6–72.5)	5.9 (5.01–6.12)	18.5 (18.20–20.10)	2.7 (2.5–2.7)
**3.Mutation Pos+TS<16% n =10**	**Median-range**	9.2 (05.2–12)	66.2 (47.6–80.0)	5.24 (4.69–6.12)	20.10 (15.90–28.90)	2.4 (1.6–2.8)
**4.Mutation Pos+TS>16% n=9**	**Median-range**	10.9 (10.5–12.0)	68.4 (54.2–80)	5.06 (4.75–6.00)	16.30 (13.50–17.60)	2.5 (2.0–2.8)
